# Psychiatric evaluation: an important component of multidisciplinary approach in pediatric burns

**DOI:** 10.12669/pjms.41.4.11307

**Published:** 2025-04

**Authors:** Erol Can Kulice, Emrah Senel, Elif Akcay, Gulser Senses Dinc

**Affiliations:** 1 Erol Can Kulice Department of Pediatric Surgery, Antalya Training and Research Hospital, 07100, Antalya, Turkey; 2 Emrah Senel Department of Pediatric Surgery, Bilkent City Hospital, 06800, Ankara, Turkey; 3 Elif Akcay, Department of Child and Adolescent Psychiatry, Bilkent City Hospital, 06800, Ankara, Turkey; 4 Gulser Senses Dinc, Department of Child and Adolescent Psychiatry, Bilkent City Hospital, 06800, Ankara, Turkey

**Keywords:** Acute Stress Disorders, Pediatric Burns, Psychiatric Diagnosis, Post-Traumatic Stress Disorders, Teeth Grinding Disorder

## Abstract

**Objective:**

To identify psychiatric symptoms and diagnoses in pediatric burn intensive care patients and to retrospectively evaluate their relationship with the severity of the burn injury.

**Methods:**

Eighty-five cases for whom psychiatric consultation was requested in the Pediatric Burn Intensive Care Unit, Ankara Child Health, Hematology, and Oncology Training and Research Hospital between January 2013 to January 2017 were included. The relationships between clinical data from burn assessments and psychiatric symptoms were analyzed retrospectively.

**Results:**

Psychiatric pathology was identified in 57 cases admitted to the burn intensive care unit. Among the cases with identified psychiatric pathology, it was found that 50.8 % (n=29) received a diagnosis of acute stress disorder, while 40.3 % were diagnosed with post- traumatic stress disorder (n=23). In the comparison of cases with and without psychiatric pathology, no significant relationship was found with the percentage of burn surface area. The frequency of post-traumatic stress disorder was 14 times higher in cases with teeth grinding (p=0.022).

**Conclusion:**

Psychiatric pathology can also emerge in cases with a small burn area. Therefore, the significant role of child psychiatry in the treatment of children hospitalized in burn centers should not be overlooked, and awareness of this process should be increased.

## INTRODUCTION

Burn injury can lead to significant morbidity and mortality, including both physical and psychological sequelae, with a considerable associated health-economic impact.^1^ In addition to the risk of mortality, burns have the potential to cause trauma that leads to emotional devastation. Consequently, various psychiatric symptoms may be observed in the post-trauma period in patients exposed to burn trauma. In pediatric cases, severe anxiety, post-traumatic stress disorder (PTSD), acute stress disorder (ASD), and major depressive disorder (MDD) may develop in one-third of patients immediately following the incident**.** Additionally, delirium, transient psychosis, depression, anxiety, and sleep disorders are psychiatric pathologies observed in these cases.^2^,^3^

Repeated painful procedures, limb loss, prolonged hospital stays, the nature of the trauma, and aesthetic issues are among the causes of various psychopathologies, particularly ASD and PTSD. Although data on the development of ASD in the broader population is limited, it was added to the Diagnostic and Statistical Manual of Mental Disorders, Fourth Edition (DSM-4) 20 years ago due to its association with PTSD. Later, in the Diagnostic and Statistical Manual of Mental Disorders, Fifth Edition (DSM-5) diagnostic criteria, ASD was removed from the category of anxiety disorders and placed under trauma and stressor-related disorders. The prevalence of ASD varies widely, and the diagnosis is made clinically by assessing the case’ s behavioral and emotional symptoms. Some of the symptoms include avoiding objects and places that remind them of the event, irritability, restlessness, anger and trauma-related dreams. Psychometric questionnaires, such as the Child Stress Disorders Checklist (CSDC), are used for the assessment of ASD.^4^

PTSD may exhibit symptoms such as restlessness, reliving the event, and reduced interpersonal relationships. The persistence of these symptoms for more than four weeks following the trauma, or their emergence after this period, supports the diagnosis of PTSD.^5^ In pediatric burn cases with PTSD, it has been reported that 73% experience reliving the event, while 65% exhibit mood disturbances.^6^

Although there are studies related to the psychiatric evaluation of pediatric burn injuries, the results remain uncertain due to limited patient numbers, deficiencies in study methodology, or improper structuring.^1^ The psychological trauma and psychiatric symptoms that arise in burn cases can lead to prolonged hospitalization and challenges in the continuation of burn treatment, resulting in more complex injuries and increased healthcare expenditures. As the approach to burn treatment is recognized as a holistic process encompassing both the physical and psychiatric rehabilitation of the case, it necessitates coordinated collaboration with child psychologists and child psychiatrists, as is the case in many clinics.^7^,^8^

This study aimed to demonstrate the necessity of a multidisciplinary approach in burn treatment by evaluating the relationships between psychiatric disorders and clinical symptoms in pediatric burn cases and the severity of the burns.

## METHODS

The study sample consists of pediatric cases aged 0-18 who were admitted to the burn intensive care unit of the training and research hospital between January 2013 to January 2017. The study data were collected retrospectively using records from the hospital information system and case files. Case data were reviewed retrospectively, including information on burn surface area, the symptoms for which a child psychiatry evaluation was requested after admission, whether any pathology was identified during the initial assessment, and the absence of a history of emotional or physical abuse in the patients’ medical histories. No signs of abuse were found during the physical examinations. It was noted that there were no psychiatric comorbidities in the past medical records.

### Ethical Approval:

It was approved by the Clinical Research Ethics Committee of the Ankara Child Health, Hematology, and Oncology Training and Research Hospital with decision number 2018-008; Dated: January 15, 2018.

The timing of the evaluation in relation to the day of hospitalization was also examined. Additionally, the treatment duration in the hospital and the follow-up results after discharge for the cases were evaluated. However, it was found that many cases did not attend the recommended follow-up evaluations after discharge, and these parameters were not included in the study. Between the specified dates, a total of 304 cases were admitted to the burn intensive care unit. As part of the burn treatment process, all cases were monitored daily by child development specialists and child psychologists according to our routine protocol, and diagnoses were established based on the DSM-5. The DSM-5 is a mental health diagnostic and classification guide published by the American Psychiatric Association (APA) and is widely used by mental health professionals worldwide. It includes the definition, classification, and diagnostic criteria for various mental disorders. These criteria are utilized by psychiatrists, psychologists, and other healthcare professionals in the field of mental health to assess patients’ mental states and make diagnoses.^9^

### Statistical Analysis:

The normality of the continuous variables included in the study was assessed graphically and using the Shapiro-Wilk test. Descriptive statistics for the variables are presented as mean ± standard deviation (SD) and median (minimum-maximum) values. To determine whether a correlation exists among all variables, the Spearman correlation test was applied to categorical variables, while the Pearson correlation test was used for continuous variables, and the results were presented. Cross-tabulations were created to compare the results of the psychiatric evaluation and the categorical variable of gender, providing counts (n), percentages (%), and chi-square test statistics.

A power analysis was conducted prior to the study to compare psychiatric evaluation results and burn surface area values. It was determined that at least 82 participants needed to be included in the study based on a two-group comparison model, with a minimum power of 80% and a significance level of 0.05, assuming an effect size of 0.60. The study was completed with 85 patients. The G*Power version 3.1.5 program (written by Franz Faul, University of Kiel, Germany, Copyright 1992-2012) was used for the power analysis.

In the comparison of psychiatric evaluation results with burn surface area values, an independent samples t-test was used, while the Mann-Whitney U test was applied to the timing of diagnosis values. Additionally, non-parametric variance analysis using the Kruskal-Wallis test was conducted for the comparison of these values.

Potential risk factors associated with PTSD were examined using multivariable logistic regression analysis. Results are presented as odds ratios (Exp(B)) with 95% confidence intervals. Statistical analyses and calculations were performed using IBM SPSS Statistics version 21.0 (IBM Corp. Released 2012. IBM SPSS Statistics for Windows, Version 21.0. Armonk, NY: IBM Corp.) and MS Excel 2007. A statistical significance level of p < 0.05 was considered significant.

## RESULTS

During treatment, following the observation of various symptoms such as restlessness, withdrawal from communication, and teeth grinding, a child psychiatrist, evaluation was requested for 85 of the cases. After examination by child psychiatrist, the cases were divided into two groups: 28 cases with no identified psychiatric pathology (Group-I) and 57 cases with identified psychiatric pathology (Group-II). Among the cases with identified psychiatric pathology, it was found that 50.8% (n=29) received a diagnosis of ASD, while 40.3% were diagnosed with PTSD (n=23). The average time to diagnosis after admission for patients diagnosed with ASD was 6.10 ± 2.76 days, whereas the average time to diagnosis after admission for those diagnosed with PTSD was 34.57 ± 5.26 days. A statistically significant difference was observed between the time to diagnosis for individuals with ASD and those with PTSD (z=6.156, p<0.001).

Among individuals with psychiatric disorders, restlessness was observed in 35.1% (n=20) and withdrawal in 26.3% (n=15) as reasons for consultation, whereas among individuals without psychiatric disorders, restlessness was detected in 39.3% (n=11) and agitation in 25.0% (n=7). A statistically significant difference was found in consultation reasons based on the presence of psychiatric disorders (χ²=20.057, p=0.005) (Table-I).

**Table-I:** Comparison of Consultation Reasons in Patients with Psychopathology.

	PSYCHOPATHOLOGY			
	Group-I (n=28)	Group-II (n=57)	χ^2^	p
	n (%)	n (%)		
Consultation Reasons				
Agitation	7 (25.0)	8 (14.0)	*χ^2^*=20.057	0.005
Teeth grinding	0 (0.0)	5 (8.8)		
Lip biting	1 (3.6)	1 (1.8)		
Restlessness	11 (39.3)	20 (35.1)		
Reduced relationships	3 (10.7)	15 (26.3)		
Recreational drug use	4 (14.3)	0 (0.0)		
Sleep disorder	2 (7.1)	4 (7.0)		
Others	0 (0.0)	4 (7.0)		

*χ^2^*: Ki kare Test

According to the results of the psychiatric evaluation, no statistically significant difference was found between diagnosed or undiagnosed and the burn surface area (p>0.05) (Table-II). Additionally, no statistically significant difference was observed between the subgroups of psychiatric disorders and the burn surface areas of the individuals (p>0.05) (Table-III).

**Table-II:** Comparison of psychiatric consultation results and burn percentage values.

	Psychiopatology					
	Group-I (n=28)		Group-II (n=57)		Test Statistics	
	Average±SD	Median (min-max)	Average±SD	Median (min-max)	t; z	p
Burn Percentage	30.43±13.27	28.0 (6-50)	35.04±14.41	35.0 (10-70)	t=1.421	0.159

t: Independent Samples T-Test z: Mann Whitney U Test

**Table-III:** Comparison of burn percentage values according to subgroups of psychiatric disorders.

	Psychiopatology subgroups and burn percentage				
	ASD (n=29)	PTSD (n=23)	OTHERS (n=5)	Test statistics	
	Average±SD Median (min-max)	Average±SD Median (min-max)	Average±SD Median (min-max)	χ^2^	p
Burn Percentage	36.93±15.82	33.43±13.98	31.40±5.03	*χ^2^*=0.578	0.749
	35.0 (11-70)	34.0 (10-70)	34.0 (25-36)		

*χ^2^*: Kruskal Wallis Test

To determine whether a correlation exists among all variables, the Spearman correlation test was applied to categorical variables, while the Pearson correlation test was used for continuous variables. A positive correlation was observed between the symptom of reduced interpersonal relationships and the diagnosis of ASD (r=0.229, p=0.035). Additionally, a significant relationship was found between PTSD and teeth grinding (r=0.246, p=0.023).

The results of the multivariable logistic regression model investigating the effects of age, gender and PTSD on the symptom of teeth grinding were evaluated. According to the results of the multivariable logistic regression analysis, the risk of developing PTSD was found to be 14.210 times higher in individuals with teeth grinding compared to those without (OR: 14.210, p=0.022) (Table-IV).

**Table-IV:** Potential risk factors associated with PTSD in the multivariable logistic regression model.

Variables	β	SD	Wald	p	Exp(B)	95% Confidence Interval for exp(B)	
						Belove	Above
Constant	-1.833	0.739	6.160	0.013	0.160		
Age	0.060	0.065	0.851	0.356	1.062	0.935	1.206
Gender	0.311	0.543	0.328	0.567	1.365	0.471	3.962
Teeth grinding	2.654	1.158	5.256	0.022	14.210	1.470	137.386

Or: Odds Ratio

## DISCUSSION

In our study, psychiatric pathology was identified in 65% of the cases that underwent psychiatric evaluation. Woolard et al. noted in their review that the risk of psychiatric pathology following pediatric burn injuries is significantly higher than in the general population, and that psychiatric treatment represents an important area that requires focus in the near future. ASD is the most commonly observed psychiatric disorder in patients with severe burns, followed by PTSD. Theoretically, ASD typically manifests between one to four weeks post-trauma, while PTSD develops after four weeks following the traumatic event. The timing of diagnoses in cases that underwent psychiatric evaluation and received these diagnoses is consistent with the literature.^2^,^10^-^12^ In both ASD and PTSD, symptoms such as involuntary re-experiencing of the event, restlessness, reduced interpersonal relationships, as well as dissociative symptoms, are observed. The literature reports that one of the frequently encountered psychiatric symptoms following trauma is reduced interpersonal relationships and restlessness.

We observed that reduced interpersonal relationships and restlessness are among the primary reasons for psychiatric evaluation, and that the incidence of ASD is increased in these cases. Therefore, we recommend that psychiatric assessments be conducted for individuals exhibiting symptoms of reduced interpersonal relationships. Similar clinical outcomes have been reported in the literature.^2^,^3^,^13^-^17^

Although the percentage of burns was found to be higher in individuals without psychiatric pathology, no statistically significant difference was identified between the mean percentages of burns in those with psychiatric pathology and those without. Therefore, it was concluded that the percentage of burns does not have a significant effect. For example; MDD is typically expected to occur after severe burns; however, it is also frequently observed in patients with minor burns.^18^-^20^ In a study by Cui L, et al, it was noted that multiple factors and hypotheses are recognized in the pathogenesis of MDD. According to the Cytokine Hypothesis, one of these theories, it is suggested that sudden changes in the levels of interleukins and pro-inflammatory cytokines play a role in the development of MDD.^21^ It was evaluated that the uncontrolled and rapid release of cytokines and other inflammatory mediators from the burn area could contribute to the development of MDD through this mechanism.

In a study by Mann SK. et al, childhood traumas were identified as the primary risk factor for the development of PTSD. Other factors that increase this risk include the severity of the trauma and the reminders of the event’s details during the post-traumatic period.^22^ In burn cases, repetitive painful procedures during treatment, as well as conscious or unconscious reminders of the event’s details through any means, may pose a risk for the development of PTSD, even in cases with small burn areas. To reduce this risk, it was concluded that appropriate conditions should be created to ensure effective pain management (sedation during wound care, post-operative analgesic treatment and the involvement of professional wound care staff.) and that families should receive sufficient guidance from child development specialists and child psychologists on how to communicate with the patient during the post-traumatic period.

In the study introduced by Karaçetin G. et al., the frequency of psychiatric pathology in burn cases was reported as 61.3%. The researchers highlighted a statistically significant relationship between the burn percentage, the number of burned areas, and the emergence of psychiatric pathology.^3^ The differing results in the two studies may stem from the limited sample sizes. We believe that multicenter studies with larger patient populations could yield more accurate results. Teeth grinding can be classified into two categories based on its occurrence during sleep or wakefulness. Sleep teeth grinding in particular, is prevalent among children. It can manifest in a variety of conditions, including psychiatric pathologies resulting from chronic stress and temporomandibular disorders. Teeth grinding tends to be more severe during periods of increased stress and anxiety. This condition, which can lead to damage of the enamel, is commonly observed in individuals with PTSD, anxiety disorders, and elevated levels of stress.^23^-^25^ Therefore, we believe that conducting a psychiatric evaluation would be beneficial when symptoms of teeth grinding are observed.

Our study is one of the few that provides a detailed analysis of psychiatric evaluation findings during the treatment of pediatric burn cases and examines their relationship with the percentage of burns. The emergence of psychiatric pathologies independent of burn percentage highlights the necessity of monitoring the mental health of burn patients. In addition to hospitalized burn patients, the mental health of outpatients should also be monitored, making this an important area for further research.

### Conflict of Interest:

A primary limitation of our study is the lack of early follow-up for patients who exhibited symptoms and received diagnoses, leading to an inability to evaluate the results of control examinations. We do not have data on long-term post-discharge follow-up for patients. Another limitation is the absence of a specific algorithm for psychiatric approaches in the care of burn patients, which is due to the recent development of such approaches. Additionally, insufficient studies and patient numbers represent another shortcoming. More studies with a larger number of patients could lead to more reliable results.

## CONCLUSIONS

1. Psychiatric symptoms and pathologies are common issues during the treatment of pediatric burn cases, and the emergence of these pathologies in burn patients is independent of the percentage of burns.
2. The need for psychiatric consultation emerged in more than a quarter of the patients, with psychiatric pathologies identified in two-thirds of these consultations. It is essential to address these issues with careful consideration as part of a multidisciplinary management approach.
3. It is crucial for the teams involved in the treatment of burn cases to follow closely the emotional state of children and to intervene promptly as part of the treatment process.
4. It should be kept in mind that the presence of reduced interpersonal relationships may increase the risk of developing ASD, while the presence of teeth grinding may indicate a higher risk of developing PTSD.
5. The prevalence, comorbidity, treatment delays, and severity of ASD and PTSD should be taken into account, and it should be emphasized that child psychologists and child psychiatrists are crucial members of the treatment team.
6. The management of pediatric burns should be conducted with a multidisciplinary approach. One of the most important disciplines in this regard is child psychiatry, and it is crucial to raise awareness about this issue.
